# Effects of thinning on soil nutrient availability and fungal community composition in a plantation medium-aged pure forest of *Picea koraiensis*

**DOI:** 10.1038/s41598-023-29498-9

**Published:** 2023-02-13

**Authors:** Zhao Caihong, Su Nier, Wang Hao, Xing Honglin, Shen Hailong, Yang Ling

**Affiliations:** State Key Laboratory of Tree Genetics and Breeding, School of Forestry, Northeast Forestry University, Harbin, 150040 China

**Keywords:** Forest ecology, Symbiosis

## Abstract

Thinning is an important silvicultural practice for improving the productivity and wood production in plantation forest. Different intensities of thinning management can affect tree growth and alter soil nutrient effectiveness, thus affecting soil fungal community structure and diversity. Our objective is to determine the soil factors and their regulatory mechanisms that influence stand growth by thinning, and to provide data to support the establishment of large diameter timber cultivation technology for *Picea koraiensis*. In this study, we conducted medium- and high-intensity thinning in 43a *P. koraiensis* plantation middle-aged forests and investigated the growth indexes, soil physicochemical properties, and fungal community diversity in rhizosphere and non-rhizosphere soils of the stands after thinning at different densities (904 plants/ha for control, 644 plants/ha for 30% thinning intensity, and 477 plants/ha for 50% thinning intensity). The results showed that all growth indicators (annual growth of tree height, diameter at breast height, height under live branches and crown width) of the plantation after high-intensity thinning (477 plants/ha) were higher than those of the control (no thinning, significant) and medium-intensity thinning (644 plants/ha). Mycorrhizal infection rate was higher at the beginning of the growing season than at the end of the growing season, and increased slightly with decreasing stand density. Compared to the control, all medium- and high-intensity thinning treatments significantly improved soil nutrient content (*P* < 0.05), including total carbon, total nitrogen, total phosphorus, total potassium, Available phosphorus and Available potassium. Fungal diversity was higher but lower in abundance than the control in both rhizosphere and non-rhizosphere soils after thinning. The number of OTUs and fungal richness and diversity indices of non-rhizosphere soil fungi were higher than those of rhizosphere soil fungi. In conclusion, this study provides new evidence that reasonable intercalation can increase the radial and vertical growth of *P. koraiensis* plantation forests and promote the diversity of subsurface soil fungal communities. It is shown that thinning intensity regulates biogeochemical cycles in *P. koraiensis* plantation ecosystems by affecting soil nutrients and fungal community structure. Therefore, 50% thinning intensity can be used to increase timber production in plantation forests during large diameter timber cultivation of *P. koraiensis* and improve predictions associated with achieving long-term forest management strategies.

## Introduction

Compared to natural forests, planted forests are more prone to fire and other natural disasters due to their over-cultivation^1^. At the same time, it appears as though nutrient cycling and material flow in plantations are locked in a vicious circle, with nutrients in the litter layer unable to be input into the soil surface layer as forest age increases^2,3^. Thus, improving the quality of plantation and the structure of stands through thinning (density regulation) is critical for forestry production^4,5^. After thinning, forest growth improved significantly due to increased availability of nutrients such as nitrogen, phosphorus, and potassium and decreased competition for available resources^6–8^. On the one hand, the formation of microclimates following thinning would provide sufficient light. Increased surface temperature and humidity would accelerate the decomposition of litter on the ground, increasing the nutrient content of litter transported to the ground^1,9–12^ and thereby providing more effective environmental conditions for the geochemical cycle^10^. On the other hand, environmental changes following thinning altered the composition of the soil microbial community, resulting in distinct responses of the rhizosphere soil microbial community involved in the forest nutrient cycle and mycorrhizal infection of forest trees^13–15^. However, previous studies have been limited to aboveground plant-related changes in stand structure, and feedback mechanisms on above- and below-ground components from various thinning intensities are unclear.

As critical biological components of forest ecosystems, microorganisms are responsible for connecting the aboveground and underground components of forest systems^16^. Microorganisms in the soil, particularly fungal communities, are highly vulnerable to environmental changes^17^. Many studies have found that when soil nutrients change, fungal communities compete to balance the nutrients required in the host rhizosphere, altering their effectiveness in energy flow and the nutrient cycle and indirectly affecting the growth status of aboveground trees^18,19^. However, it is still uncertain whether the fungal community changes with soil nutrients in response to different intensities of thinning. Simultaneously, soil organic matter decomposition is constrained by microorganisms’ use of nitrogen. The host competes for nitrogen with microorganisms that live freely in the soil via fungal symbionts (mycorrhizae)^20^. Thus, soil nitrogen content indirectly reflects the diversity of microbial communities^21^. However, how thinning affects the relative abundance of mycorrhizal fungi and microorganisms remains inconclusive. Iwaoka^22^ and Bahnmann^23^ reported that the pH of the soil did not affect the availability of soil nutrients and, thus, the fungal community in the soil. However, there are still few reports on whether thinning responds to soil pH, which in turn affects soil nutrients and fungal communities, and the link between soil and microorganisms remains unclear. Previously, traditional culture methods were used for microbial community studies, which severely limited our understanding of community diversity and structure. In recent years, with the development of molecular biology, high-throughput sequencing technology has been widely used, allowing us to have a more comprehensive understanding of soil fungal communities.

*Picea koraiensis* is a species of evergreen trees found primarily in northeastern China, Korea, and Russia's Far East, having undergone heavy logging in the past, most of the existing forests are medium and young forests restored after logging and existing plantations. *P. koraiensis* is an important tree species for afforestation in Northeast China and can also be used as an ornamental in gardens. It grows slowly at first but rapidly after ten years, during which it undergoes a vigorous growth period. With proper tending management and planting density, the maximum annual growth of *Larix gmelinii* (Ruprecht) Kuzeneva can approach the peak and has a long-term exuberant growth period. Because current research on *P. koraiensis* is primarily focused on seedlings^24,25^, afforestation^26,27^, and so on, *P. koraiensis* cultivation technology and management methods have not yet reached an optimal level. Establishing a rational and effective tending management mechanism is necessary to allow *P. koraiensis* to exhibit its good ecological characteristics and cultivate high-quality large-diameter timber. Because *P. koraiensis* is grown for its large diameter, it is critical to research how to control stand density.

From this perspective, in this study our current research objective was to explore the changes in stand growth status, mycorrhizal infection rate and fungal community composition in *P. koraiensis* plantations induced by thinning. Specifically, we addressed the following questions: (1) How do different thinning treatments affect the growth status and mycorrhizal infection rate of a stand? (2) Do different soil nutrients respond to changes in thinning intensity? (3) How does thinning affect fungal community composition by altering soil nutrients? Our results are expected to provide a scientific basis for sustainable development of plantation forest ecosystems and large diameter timber cultivation techniques.

## Methods and materials

### Experimental site

**Figure 1:**
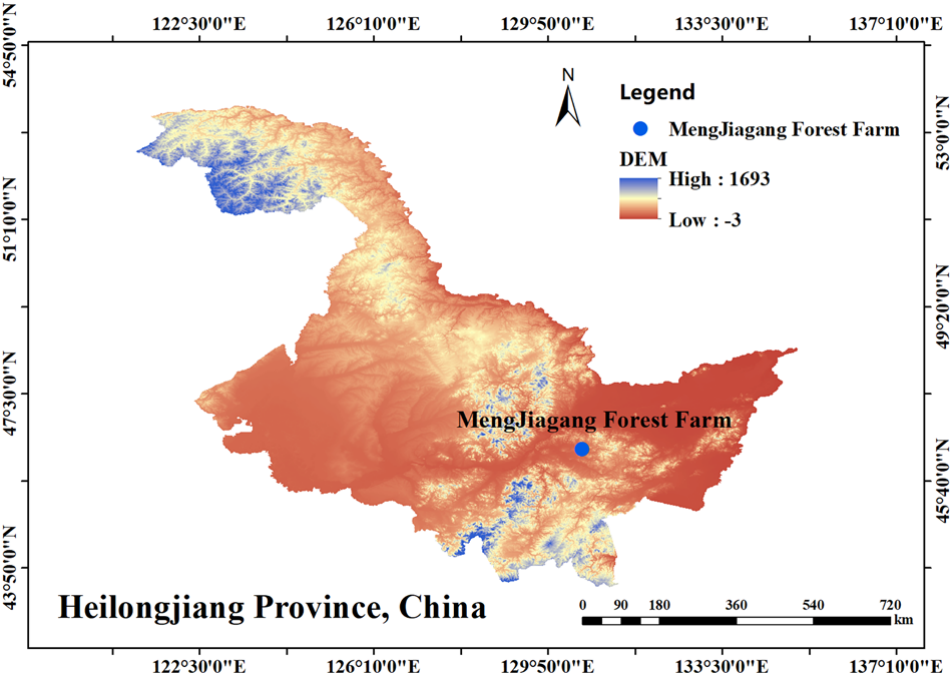
Study area location in Mengjiagang, Heilongjiang Province of China. Note: this map was generated using the ArcGIS10.8 software from American ESRI Company.

The research site was located in the MengJiagang Forest Farm (Fig. 1), Jiamusi Forestry Bureau, Heilongjiang Province, China (130°32 ″42 ″~ 130°52″ 36 ″E, 46°20″ 16 ″~ 46°30 ″50″ N). It is located at the western foot of Wanda Mountain and is dominated by low hills, with an altitude of 170–575 m, and it belonged to the continental monsoon climate of the middle temperate zone. The average annual temperature was 2.7 °C. The highest temperature was 35.6 °C, and the lowest temperature was − 38.6 °C. Station meteorological data from China Meteorological Data Network, was collected from Jiamusi weather station (site 50,873). The average annual rainfall was 550 mm, the frost-free period was 120 days, and the annual sunshine duration was 1955 h. The soils are mainly dark brown loam.

### Sample plot setting and investigation

The investigated stand is in one of the MengJiagang Forest Farm's 50 forest classes and has a forestation age of 43a and a forested area of 375 hectares. The stand was grown and cut twice in 2005 and 2010, and the stand density was uneven. Three thinning intensities of plant numbers were determined based on the current stand density distribution in 2017: control (904 plants/ha), 30% thinning intensity (644 plants/ha), and 50% thinning intensity (477 plants/ha). Each treatment was repeated three times on a fixed sample plot of 30 m × 30 m. Table 1 summarizes the primary conditions affecting the physical and chemical properties of the soil in the sample plot. Following thinning at the end of 2017, each plot's management measures remained consistent. At the end of the growing seasons in 2017 and 2018, we measured the tree height, diameter at breast height (DBH), crown width, and live branch height of *P. koraiensis* trees in the sample plots. In 2018, soil and root samples of *P. koraiensis* trees were taken at the start and end of the growing season to determine the soil's physical and chemical properties and mycorrhizal infection rate. In July, samples from the rhizosphere and the non-rhizosphere soils of *P. koraiensis* trees were taken to find out the soil's fungal community diversity and composition.

**Table 1:** Physical and chemical properties of the soil in the study sample plot.

Soil depth (cm)	pH	TC (mg/kg)	TN (mg/kg)	TP (mg/kg)	TK (mg/kg)	AP (g/kg)	AK (g/kg)
0–20	5.52	0.39	4.69	11.95	5.70	53.13	92.05
20–40	5.50	0.27	3.29	8.97	3.75	54.25	75.72

### Investigation on forest growth

All trees in the sample plots were checked for each wood, and the tree height (m) was measured with a measuring tape; the tree diameter at breast height (cm) was measured at 1.3 m from the ground with a diameter at breast height ruler; and the height under live branches and crown width (m) were measured with a meter ruler.

### Collection of root sample and determination of mycorrhizal infection rate

The collection of root and soil samples was approved by Mengjiagang Forest Farm. And complied with relevant institutional, national, and international guidelines and legislation. Five target trees (*P. koraiensis*) were randomly selected in the sample plot, and the root segments contained in the 0–20 cm soil layer were dug with a spade at the position about half a meter away from the trunk in the extension direction of the root system at the base of each target tree. After mixing, the mixture was put into a liquid filled with FAA stationary phase, and then put into a cold storage box at 1–3 °C. Fifty roots were chosen randomly from each plastic bottle containing Grade 1 FAA root fixative. Phillips and Hayman's staining methods were used to observe roots with hypha wrapping at the root tip and thick and round roots with a mycorrhizal infection under a microscope (10×). The rate of mycorrhizal infection was calculated using the following formula:

$$ {\text{Mycorrhizal}}\,{\text{infection}}\,{\text{rate}}\, = \,{\text{number}}\,{\text{of}}\,{\text{infected}}\,{\text{root}}\,{\text{ segments}}/{\text{total}}\,{\text{ number }}\,{\text{of }}\,{\text{detected }}\,{\text{root }}\,{\text{segments}}\, \times \,{1}00\% $$

### Collection of soil samples and determination of elemental composition

The five-point method was used to select the soil with a depth of 0–20 cm and 20–40 cm in the sample plot (Five samples in each plot were homogenized into a composite sample). After removing the visible roots and crop residues, the soil was merged together to make composite samples on the same piece of land. The samples were divided into two parts by the four-part method. One part was put into a refrigerator at 4 °C for preservation. The ammonium and nitrate nitrogen contents were measured using a continuous flow analyzer after being extracted with potassium chloride solution. The other was air-dried and tested for total soil carbon, total nitrogen, total phosphorus, total potassium, available phosphorus, and available potassium contents, as well as soil pH. The soil pH was determined using a pH meter when the soil-to-deionized-water ratio was 2.5:1. The total carbon and total nitrogen contents of the soil was determined using an elemental analyzer. After the soil was leached with perchloric acid and concentrated sulfuric acid, the total phosphorus content was determined by the molybdenum-antimony anti-spectrophotometer method, and the total potassium content was measured by a flame photometer. After the soil was extracted by double acid leaching, the available phosphorus content was determined by a molybdenum-antimony anti-spectrophotometer. The soil was leached with neutral ammonium acetate, and the total potassium content was determined by a flame photometer.

### Determination of fungal communities in rhizosphere and non-rhizosphere soil

The rhizosphere and non-rhizosphere soil samples were collected by the five-point method in the sample plot in July 2018 and quickly put into an incubator and sent back to the laboratory for freezing at − 80 °C. The obtained samples were subjected to fungal DNA extraction and detection by GENE DENOVO Biology Co., Ltd. (Guangzhou, China). After genomic DNA was extracted from the soil sample, the ITS2 region of ITS rDNA was amplified by using specific primers with a barcode. The primer sequences were KYO2F: GATGAAGAACGYAGYRAA; ITS4R: TCCTCCGCTTATTGATATGC. The PCR amplification products were then recovered by gel cleavage and quantified using a QuantiFluorTM fluorometer. The amplification products were mixed in equal amounts, connected to the sequencing adaptor, and sequenced on an Illumina HiSeq 2500 platform by paired-end sequencing.

### Statistical analysis

Data were processed using Microsoft Excel 2010 for forest growth conditions, and one-way analysis of variance (ANOVA) was performed on the data using IBM SPSS Statistics 21 statistical analysis software to test soil physicochemical properties of different treatments and fungal richness index and diversity index among different samples, and multiple comparison analysis (LSD) was performed at 95% confidence level. Homogeneity of variance was assessed by Levene’s test for equality of variance (*P* > 0.05). Plotting was performed with SigmaPlot 12.5 software (SYSTAT Inc.). The calculated OTUs were used to make a Venn diagram through the Venn Diagram in R (3.0.3) language and to identify OTUs common and unique to the samples. α Diversity and NMDS analyses were similarly calculated and plotted by rooting the inter-OTUs. One-way ANOVA was performed using IBM SPSS Statistics 21 statistical analysis software. Community composition and structure at all levels were statistically analyzed according to OTU clustering, and abundance heat maps were plotted using PICRUSt software.

### Ethical approval

We statement that this study complies with relevant institutional, national, and international guidelines and legislation, and permissions for collecting the plant and seed specimens has been obtained.

## Results

### Effect of stand density on the annual growth of forests

The annual growth of each growth index of *P. koraiensis* stands increased with the decrease in the stand density (Table 2). The annual growth of DBH of the two thinning intensities was 21.98% and 47.25% (*P* < 0.05), respectively, higher than that of the control, and the annual growth of tree height was 65.52% and 66.02% (*P* < 0.05), respectively, higher than that of the control. The annual growth of height under the live branch was 65.28% and 81.88% (*P* < 0.05), respectively, higher than that of the control. The annual growth of the crown width in the east and the west was 35.14% and 61.90% (*P* < 0.05), respectively, higher than that of the control. The annual growth of the crown width in the south and the north was 52.27% and 66.67% (*P* < 0.05), respectively, higher than that of the control.

**Table 2:** Annual growth of *Picea koraiensis* under different density intensities.

Thinning intensity	Stand density (plant/ha)	DBH (cm)	Hight (m)	Live height (m)	Crown (m)	
					East–west	South-north
CK	904	0.91 + 0.04b	0.70 + 0.11c	0.25 + 0.14c	0.24 + 0.01c	0.21 + 0.49b
30%	644	1.11 + 0.12a	2.03 + 0.57b	0.72 + 0.13b	0.37 + 0.04b	0.44 + 0.84a
50%	477	1.34 + 0.22a	2.06 + 0.68a	1.38 + 0.14a	0.63 + 0.06a	0.63 + 0.17a

Values followed by a different lowercase letter are significantly different at the 0.05 level.

### Effect of stand density on the infection rate of fine root mycorrhizal fungi

**Figure 2:**
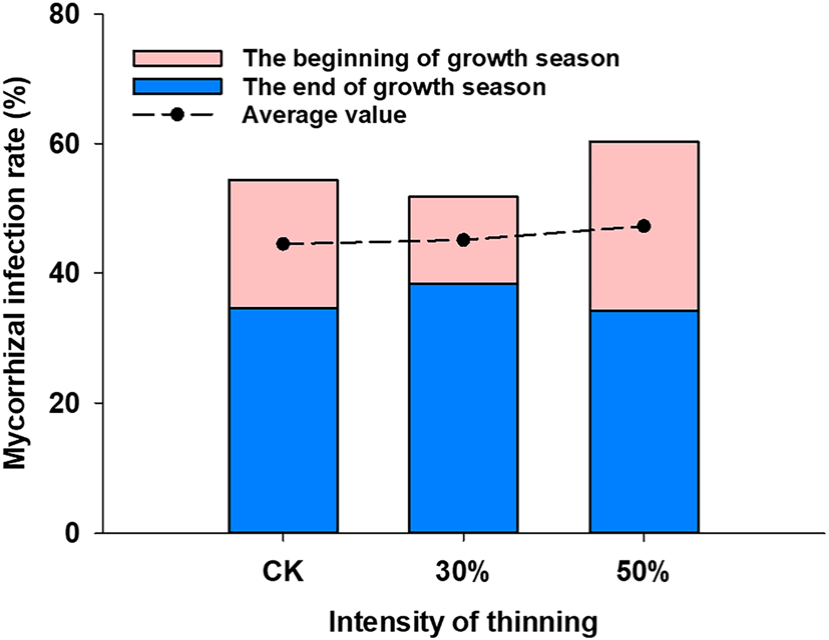
Comparison of mycorrhizal infection rate at the beginning of the growing season and at the end of the growing season between *Picea koraiensis* stands with different densities.

The mycorrhizal infection rates at the beginning and end of the growing season in stands with different densities are shown in Fig. 2. Mycorrhizal infection showed different degrees of variation at the beginning and at the end of the growing season as the stand density decreased. At the beginning of the growing season, the mycorrhizal infection rate was higher at 50% thinning intensity (477 plants/ha) than the control and 30%, while the difference between treatments at the end of the growing season was not significant. However, in general, 50% thinning intensity increased the rate of mycorrhizal infection of fine roots.

### Effect of stand density on soil physical and chemical properties

**Figure 3:**
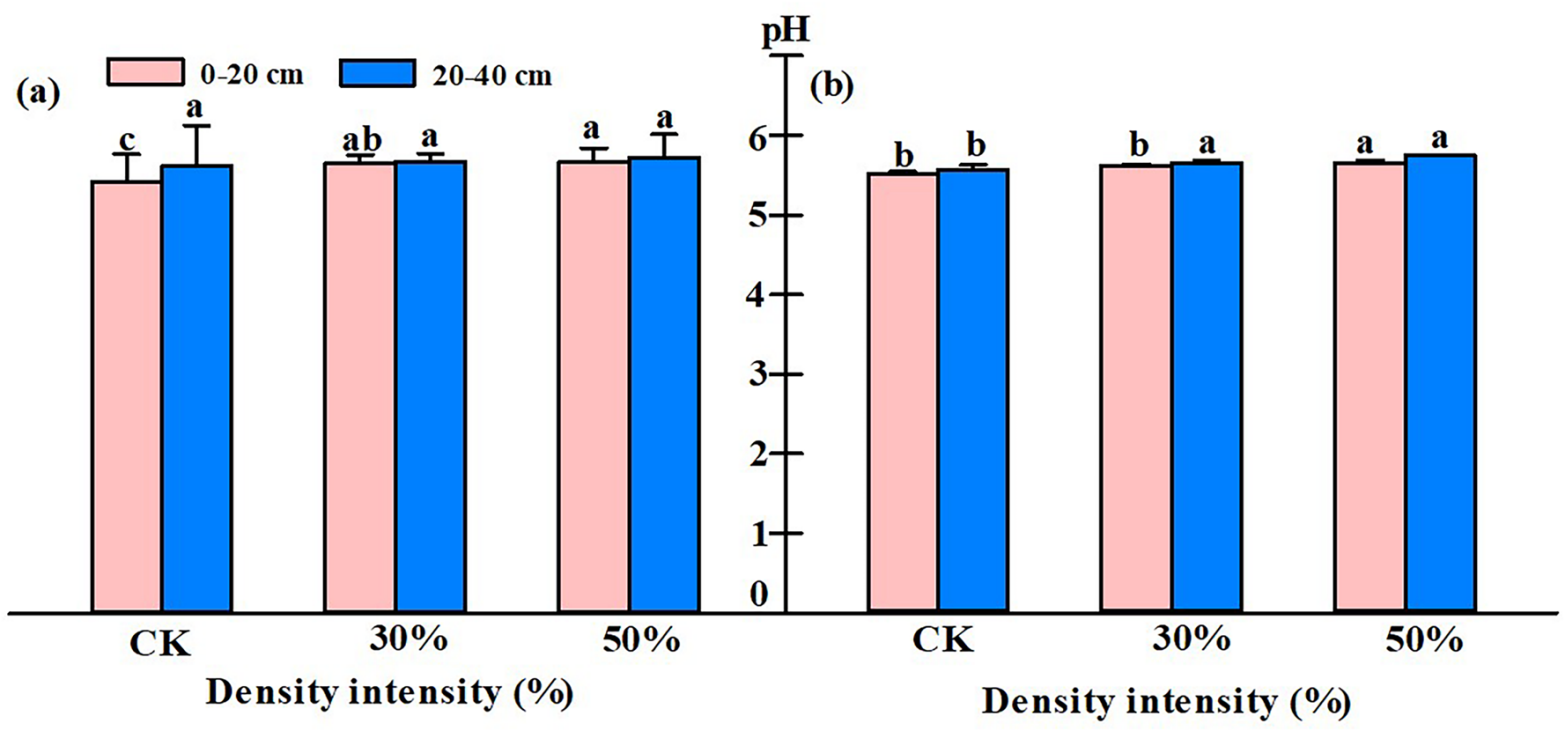
Comparison of pH values of different soil layers of *Picea koraiensis* plantations with different densities at the beginning (**a**) and the end (**b**) of the growing season.

After thinning with two intensities, the soil pH had no significant difference between the beginning and the end of the growing season and was weakly acidic. The value increased with the decrease in stand density (Fig. 3). The acidity of the 0–20 cm soil layer was higher than that of the 20–40 cm soil layer.

The changing trends of soil nutrient content between the beginning and end of the growing season were different (Table 3). At the beginning of the growing season, the soil total carbon and total nitrogen contents in the two soil layers of the two stands under different thinning treatments were higher than those of the control, and the soil total carbon content in the surface layer (0–20 cm) was significantly different from that of the control (*P* < 0.05). The C/N ratios of the top (0–20 cm) and bottom (20–40 cm) soils under the two thinning treatments were the same but lower than that of the control. The total phosphorus and total potassium contents in the surface and deeper soil layers of the two stands under different thinning treatments were significantly higher than those in control (*P* < 0.05). Among them, the stand with 50% thinning intensity (477 plants/ha) had the highest total phosphorus content in both soil layers (80.82% and 86.67% higher than that of the control, respectively). For total potassium content in the soil, it was highest in the topsoil (0–20 cm) of the 30% thinning (644 plants/ha) stand (44.86% higher than that of the control) and the subsoil of the 50% thinning (477 plants/ha) stand (134% higher than the control). With the decrease of stand density, the available phosphorus and potassium contents in both soil layers were significantly increased, while the available nitrogen content was significantly decreased (*P* < 0.05).

**Table 3:** Comparison of soil chemical properties of *Picea koraiensis* plantation between the beginning and the end of the growing season in stands with different densities.

Thinning intensity (%)	Stand density (plant/ha)	Growing season	Soil layer (cm)	Total carbon (TC)	Total nitrogen (TN)	Total phosphorus (TP)	Total potassium (TK)	Available phosphorus (AP)	Available potassium (AK)	Ammonium nitrogen (NH^4+^-N)	Nitrate nitrogen (NO_3_-N)
CK	904	Begin	0–20	0.30 ± 0.04b	3.13 + 0.56a	0.73 + 0.05c	1.07 + 0.06c	6.02 + 0.42c	256.77 + 1.11c	3.68 + 0.08a	9.95 + 0.97a
			20–40	0.15 + 0.03a	1.48 + 0.38a	0.45 + 0.03c	0.44 + 0.01c	4.47 + 0.15c	230.01 + 2.44c	3.41 + 0.10c	3.49 + 0.62a
		End	0–20	0.16 + 0.04a	1.89 + 0.25a	0.51 + 0.11a	1.59 + 0.13b	5.79 + 0.62c	126.67 + 0.33c	3.67 + 0.03a	12.54 + 0.12a
			20–40	0.11 + 0.02a	1.19 + 0.10a	0.40 + 0.08a	1.15 + 0.16c	1.47 + 0.09c	116.39 + 0.84a	3.42 + 0.10a	2.88 + 0.13a
30%	644	Begin	0–20	0.42 + 0.03a	4.82 + 0.51a	1.03 + 0.12b	1.55 + 0.13a	8,12 + 0.25b	310.80 + 1.48ab	3.41 + 0.05b	6.37 + 0.15b
			20–40	0.17 + 0.31a	1.84 + 0.44a	0.55 + 0.02b	0.88 + 0.01b	5.99 + 0.44b	261.33 + 0.22b	3.30 + 0.07b	3.44 + 0.56a
		End	0–20	0.14 + 0.01b	1.54 + 0.05a	0.47 + 0.11a	2.29 + 0.16a	11.62 + 0.89b	203.40 + 0.57b	3.41 + 0.01b	4.67 + 0.16c
			20–40	0.12 + 0.01a	1.18 + 0.03a	0.38 + 0.06a	1.21 + 0.25a	4.54 + 0.25b	135.86 + 0.61b	3.29 + 0.12a	1.54 + 0.09b
50%	477	Begin	0–20	0.43 + 0.05a	5.05 + 0.72a	1.32 + 0.01a	1.37 + 0.07ab	16.67 + 1.13a	357.55 + 0.08a	3.43 + 0.08b	5.52 + 0.74b
			20–40	0.19 + 0.12a	1.99 + 0.17a	0.84 + 0.03a	1.03 + 0.02a	11.86 + 0.49a	296.02 + 1.59a	3.24 + 0.11a	3.20 + 0.37a
		End	0–20	0.21 + 0.02a	2.39 + 0.15a	0.58 + 0.10a	2.10 + 0.16a	16.03 + 0.44a	271.20 + 0.25a	3.43 + 0.02b	8.36 + 0.11b
			20–40	0.10 + 0.01a	1.15 + 0.11a	0.41 + 0.06a	1.17 + 0.01b	5.69 + 0.09a	154.36 + 0.61c	3.25 + 0.05a	2.52 + 0.16a

Values followed by a different lowercase letter are significantly different at 0.05 level.

At the end of the growing season, the contents of total carbon, total nitrogen, total phosphorus, and the C/N ratio in different soil layers of stands with varying densities were lower than those at the beginning of the growth season (Table 3). Compared with the control, the total nitrogen and total phosphorus contents in different soil layers of stands with different densities were not significantly different. The total carbon content in the topsoil (0–20 cm) of the 30% thinning (644 plants/ha) stand was considerably lower than that of the control (904 plants/ha), with no significant difference for the rest. The total potassium content in different soil layers of stands with varying densities was higher than that at the beginning of the growing season. The two thinning treatments increased the total potassium content in the soil, and the total potassium content in the subsoil (20–40 cm) was higher than that in the surface soil layer (0–20 cm). Among the stands with different densities, the total potassium content in the two soil layers of the 30% thinning stands (644 plants/ha) was higher than those of stands with other densities (44.03% and 5.21% higher than those of the control, respectively, *P* < 0.05). With the decrease of stand density, the available phosphorus and potassium contents in the soil increased significantly (*P* < 0.05). Among them, the available phosphorus content at the end of the growing season was substantially higher than that at the beginning of the growing season. The available potassium content was lower than that at the beginning of the growing season. The ammonium nitrogen content among the stands with different densities was not significantly different. In contrast, the nitrate-nitrogen content of the stands under two thinning treatments was significantly lower (168.52% and 50%, respectively) than that of the control.

### Effects of stand density on fungal abundance and diversity in rhizosphere and non-rhizosphere soils

The microbial abundance and diversity indices of the rhizosphere and non-rhizosphere soils of *P. koraiensis* at different densities are shown in Table 4. The Sobs index, the Chao index, and the Ace index representing fungal richness, and the Shannon index and the Simpson index representing fungal diversity were all higher in the non-rhizosphere soil of stands with different densities compared to those in the rhizosphere soil. In the non-rhizosphere and rhizosphere soil, fungal Sobs index, Chao index, and Ace index of the control were higher than those of the stands under density treatments. Among them, the fungal Sobs index of the non-rhizosphere soil in the control was significantly higher than that of the stands with 30% and 50% density treatments (*P* < 0.05), and the Chao index and Ace index were considerably higher than those of the stand with 30% density treatment. They were not significantly different compared to those of the stand with 50% density treatment (*P* > 0.05). The richness indexes of the rhizosphere soil in the stands with two thinning treatments were not significantly different compared with that of the control (*P* < 0.05). The fungal Shannon index of the non-rhizosphere and rhizosphere soil in the control was lower than that of the stands with thinning treatments. The fungal Simpson index of the non-rhizosphere soil showed the same trend, but the fungal Simpson index of the rhizosphere soil in the control was lower than that of the stands under thinning treatments without significance (*P* > 0.05).

**Table 4:** Microbial abundance and diversity indices in rhizosphere and non-rhizosphere soils of *Picea koraiensis* plantations with different densities.

Items	Density intensity (%)	Stand density (plant/ha)	Richness index			Diversity index	
			Sobs	Chao	Ace	Shannon	Simpson
Non-rhizosphere soil	CK	904	706.00 ± 8.73a	890.39 ± 11.56a	872.03 ± 13.79a	5.75 ± 0.11a	0.96 ± 0.01a
	30%	644	513.67 ± 14.94c	764.56 ± 38.94b	785.83 ± 29.91b	5.92 ± 0.26a	0.96 ± 0.01a
	50%	477	618.33 ± 36.92b	846.68 ± 22.76ab	845.00 ± 23.81ab	5.91 ± 0.04a	0.96 ± 0.002a
Rhizosphere soil	CK	904	499.00 ± 20.00a	694.94 ± 41.41a	707.11 ± 47.21a	5.02 ± 0.12a	0.93 ± 0.01a
	30%	644	494.33 ± 12.60a	676.18 ± 30.28a	698.45 ± 39.22a	5.62 ± 0.34a	0.94 ± 0.01a
	50%	477	475.33 ± 23.51a	668.46 ± 5.66a	676.19 ± 12.83a	5.71 ± 0.33a	0.95 ± 0.01a

Values followed by different lowercase letters are significantly different at the 0.05 level.

### Effects of stand density on fungal community structure in rhizosphere and non-rhizosphere soils

**Figure 4:**
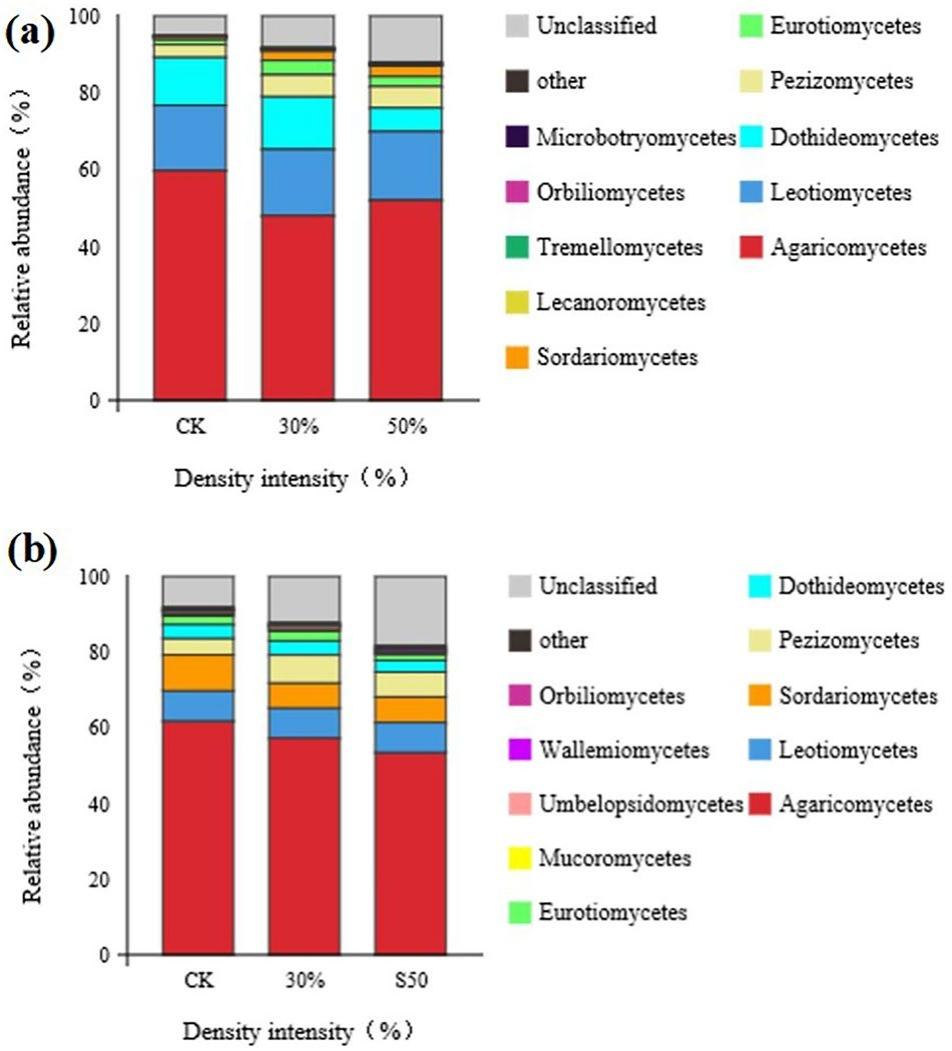
Fungal community structure distribution in the rhizosphere and non-rhizosphere soil at the class level in *Picea koraiensis* stands with different densities. (**a**) Rhizosphere soil fungi, (**b**) non-rhizosphere soil fu.

As shown in Fig. 4-a, the horizontal stacking diagram of Rhizosphere Soil Mycelia shows that, after different thinning treatments, *Agaricus* was the dominant community with the highest relative abundance. At the same time, the relative abundance in the control was higher than that in the 30% and 50% thinning treatments 24.36% and 14.77%.

The communities of Leotiomycetes, Sordariomycetes, Tremellomycetes, and other unidentified fungi increase with the decrease of stand density. The relative abundance of Dothideomycetes, Pezizomycetes, Eurotiomycetes, Lecanoromycetes, Orbiliomycetes, and Microbotryomycetes in the 30% density stand was higher than that of the control and 50% thinning stand.

The horizontal overlay of non-rhizosphere soil fungal classes is shown in Fig. 4b. Agaricomycetes had the highest relative abundance among all the fungi in the control soil (61.68%). In addition, the relative abundance of Leotiomycetes, Sordariomycetes, and Eurotiomycetes was the highest in the control, followed by the 30% thinning stand, and the lowest was found in the 50% thinning stand. The relative abundance of Pezizomycetes, Dothideomycetes, and Umbelopsidomycetes was the highest in the 30% thinning stand, while that of Wallemiomycetes, Orbiliomycetes, and other unclassified fungal communities was the highest in the 50% thinning stand.

### Analysis on the difference in soil fungal community in rhizosphere and non-rhizosphere soils between stands with different densities

**Figure 5:**
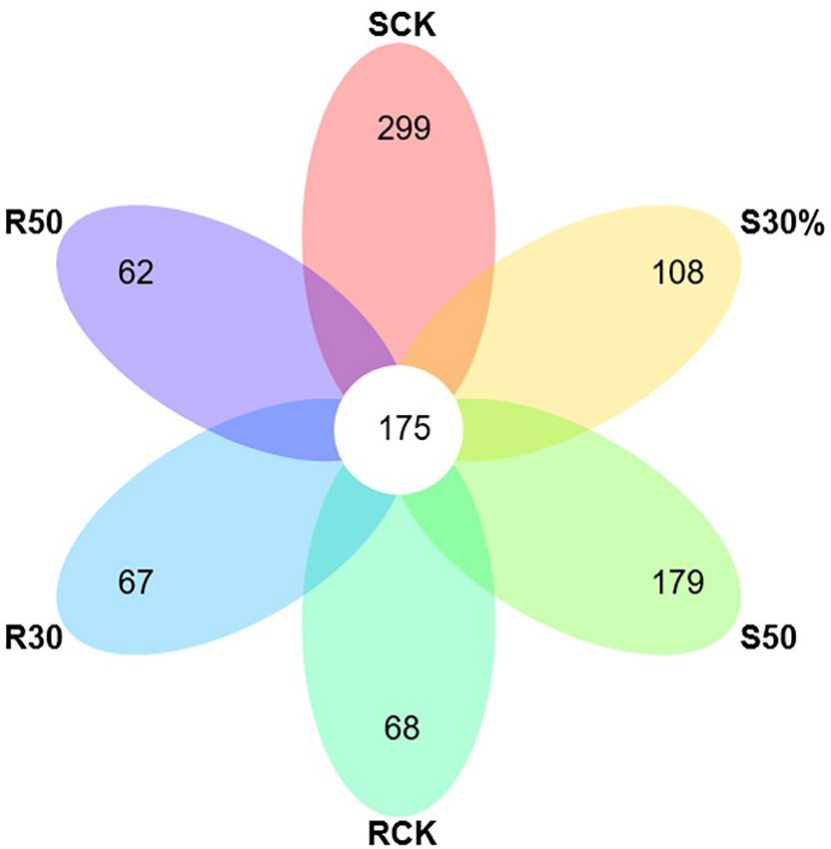
Differences in fungal OTUs between rhizosphere and non-rhizosphere soils of stands with different densities.

**Figure 6:**
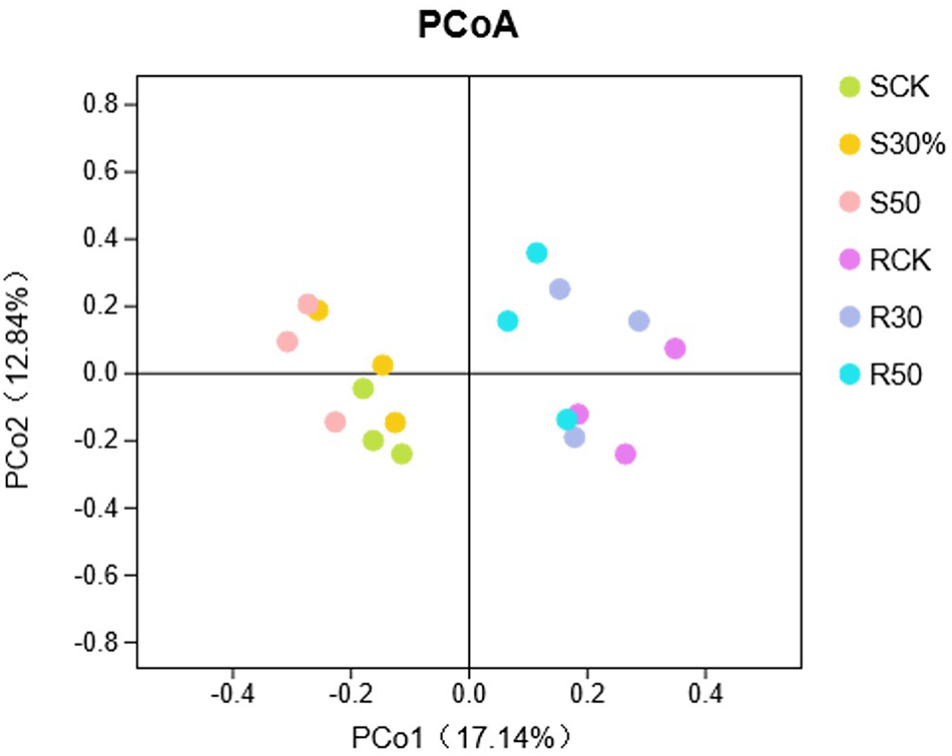
PCoA analysis of fungal communities in rhizosphere and non-rhizosphere soils of stands with different densities (S: non-rhizosphere soil fungi, R: rhizosphere soil fungi).

According to the difference in the OTUs (operational taxonomic units) between the rhizosphere and non-rhizosphere soil samples (Fig. 5), the number of fungal OTUs shared by rhizosphere and non-rhizosphere soil samples was 175. With the decrease of stand density, the number of fungal OTUs in the non-rhizosphere soil also decreased. The number of fungal OTUs in the rhizosphere soil was lower than that in the non-rhizosphere soil, and the number of fungal OTUs in the 50% thinning stand was the highest, followed by the control and the 30% thinning stand. As shown in Fig. 6, the fungal communities in non-rhizosphere and rhizosphere soils were spread out on two sides of the PCo1 spindle, with significant differences.

## Discussion

### Effects of different stand densities on the growth of *P. koraiensis* plantation

Reasonable adjustment of spatial stand structure is a meaningful way to maintain sustainable forest management^28^. Thinning could improve the overall vitality of trees, thus producing a specific feedback effect on the biological and non-biological factors of the environment^4,29^. Many studies have concluded that the stand after thinning does have growth advantages in various aspects compared with the stand with original density^30–33^. Repola^34^studied the growth status of trees after light, medium, and severe thinning in the mixed forest of *Picea abies* and *Betula pubescens* and found that different thinning intensities significantly affected the growth of trees. Severe thinning especially improved the tree thickening of *P. abies*. The results obtained from the present study were similar to those of previous authors, in that thinning treatments significantly increased the annual growth of *P. koraiensis* plantations in terms of diameter at breast height, tree height, height under live branches, and crown width. Further analysis showed that severe thinning was more beneficial to the radial and longitudinal growth of *P. koraiensis* plantation. Based on the analysis of growth habits, *P. koraiensis* grew in a relatively shade-tolerant environment. The limitation of light was not as substantial as that of shade-tolerant and non-shade-tolerant trees, so high-intensity thinning could significantly improve the growth of *P. koraiensis*^35^. The trees left after the thinning could spread out more nutrients and water, so the overall growth condition of trees with 50% thinning intensity was the best overall.

### Effects of different stand densities on soil nutrients and fine-root mycorrhizal infection rate of *P. koraiensis* plantations

The presence of forest gaps after thinning affects the microclimate, resulting in changes in surface litter decomposition rate or soil nutrient fixation and mineralization^9^. According to the nutrient status of *P. koraiensis* treated with different densities, we found that thinning treatment could improve the nutrient content of part of the soil of the *P. koraiensis* forest (Table 1). Notably, after thinning at 50% intensity, the nutrient content was significantly higher than that of the control and 30% density stand, except for the available nitrogen content. Although many studies have found that the total carbon and total nitrogen contents of forest soil after thinning decreased, both our study and Kim's study found that the pH, as well as total carbon and total nitrogen contents of the soil after thinning increased with the increase of thinning intensity^36^. The main reasons for the opposite result were that different tree species had different responses to forest gaps^37^, and the time after thinning, climate, and many other factors would affect soil carbon and nitrogen reserves^38,39^. The primary sources of phosphorus and potassium in the soil were the mineralization of litter and the microclimate in the forest gap after thinning, which would accelerate litter decomposition. Therefore, with the increase in thinning intensity, soil total phosphorus and total potassium will increase. Blanco's study also showed that thinning could increase the phosphorus and potassium content in the soil and improve the utilization efficiency of soil nutrients^40^. Our study showed that after 50% thinning treatment, the total phosphorus and total potassium contents were higher than those of the control at the beginning and end of the growing season. The available phosphorus and available potassium contents in different soil layers increased with the decrease of stand density at the beginning and end of the growing season. The soil nutrient contents under different thinning intensities of *C. lanceolata* (Lamb.) Hook*, *studied by Zhou et al.^12^ also showed that the available phosphorus and potassium contents in the soil increased after severe thinning. The transformation of organic nitrogen in soil requires the participation of soil microorganisms in the nitrogen cycle^41,42^. Our study concluded that thinning decreases the levels of soil ammonium and nitrate nitrogen, probably due to changes in the fungal community.

Mycorrhiza is an essential factor improving the mineral nutrition, growth, and development of host plants, which can significantly increase the effectiveness of plants in absorbing water and nutrients from the soil^43,44^. Sebastiana^45^ demonstrated that the rate of Mycorrhizal infection caused different responses to changes in the external environment. Early studies found that thinning was detrimental to mycorrhizal growth and may be related to a decrease in soil acidity and an increase in effective phosphorus content with increasing thinning intensity, leading to a decrease in mycorrhizal infestation rate^46^, but it has also been suggested that thinning changes the community composition of mycorrhizae^47,48^.The effect between mycorrhizal infection rate and thinning intensity was small in this study, but overall 50% thinning intensity was slightly higher than the control, thus indicating that mycorrhizae may bring about an increase in mycorrhizal infection rate due to the uptake of soil nutrients.

### Effects of different thinning densities on fungal diversity in rhizosphere and non-rhizosphere soils of *P. koraiensis* plantations

Environmental changes affect microbial diversity and community structure to a certain extent. Hawkes found that soil temperature and respiration rate after environmental changes affected fungal communities' metabolism and growth rates^49^. Our study showed that the fungal richness indices in the rhizosphere and non-rhizosphere soils after thinning were lower than those of the control (Table 4). The main reason was that the emergence of forest gaps after thinning improved the soil temperature, but climatic problems, such as continuous rain, led to excessively high soil humidity in forest land after thinning, which was not conducive to the reproduction of soil fungi^50^. Effective nitrogen cycling requires the involvement of more fungal communities, primarily due to climatic and environmental changes^51^. Our study also showed that fungal diversity in the rhizosphere and non-rhizosphere soil after thinning was higher than that in the control (Table 4). Based on the superposition diagram of fungal communities shown in Fig. 4, Agaricomycete and Leotiomycetes were the dominant communities in both the rhizosphere and the non-rhizosphere soils. After thinning, the relative abundance of these two fungal communities dropped.

### Differences in fungal communities between rhizosphere and non-rhizosphere soils

There are connections and differences between rhizosphere and non-rhizosphere soil microorganisms. The differences between them are not only affected by the environment but also by the growth status of the host and root exudates^52^. Many studies have concluded that the abundance of fungal communities in the rhizosphere soil is higher than that in the non-rhizosphere soil, mainly because the total carbon and total nitrogen contents in the rhizosphere soil are higher than those in the non-rhizosphere soil^53^. This study used PCoA to analyze the fungal communities in rhizosphere and non-rhizosphere soils under different density treatments. The results showed that the fungal communities in rhizosphere and non-rhizosphere soils were distributed entirely in different spaces (Fig. 6). However, the number and abundance index of OTUs of the non-rhizosphere soil fungi were higher than those of the rhizosphere soil fungi, which might be due to the inhibition effect of rhizosphere secretion on the growth of rhizosphere fungi^54^, which caused the activity of microorganisms attached to the rhizosphere to be lower than that of the non-rhizosphere soil fungi. According to the superposition diagram of rhizosphere and non-rhizosphere soil fungi, Agaricomycetes was the dominant community with the highest number in rhizosphere and non-rhizosphere soils. The number of Leotiomycetes and Dothideomycetes in the rhizosphere soil was higher than that in the non-rhizosphere soil. The number of Pezizomycetes and Sordariomycetes in the non-rhizosphere soil was higher than that in the rhizosphere soil.

## Conclusions

In this study, we investigated the effects of different treatments of thinning intensity on the growth status, mycorrhizal infection rate, soil nutrients and changes in fungal community structure of *P. koraiensis* plantations. The results showed that *P. koraiensis* plantations exhibited different dynamics under different intercutting management practices. The radial and longitudinal growth of *P. koraiensis* plantations increased with decreasing stand density (i.e., 50% high intensity thinning), and the height under live branch and crown width also showed the highest performance at the minimum density. The overall effect of 50% thinning intensity on mycorrhizal infection rate was slightly increased. The 50% thinning intensity of *P. koraiensis* plantations improved soil nutrient accumulation, increased soil fungal diversity, and changed soil fungal community structure more than the 30% thinning intensity. In addition, both inter- and non-inter-root soil fungal diversity were higher than the control after thinning, indicating a strong interaction between thinning and soil fungal community diversity. Fungal communities changed with soil nutrients in response to different intensities of thinning. Overall, our study confirms the importance of thinning on the growth and nutrient accumulation of *P. koraiensis* plantations, making the environment more favorable for fungal growth.

## Data Availability

Raw sequencing data have been deposited in the Sequence Read Archive (SRA) of the National Centre for Biotechnology Information (NCBI) under BioProject ID PRJNA849319 (The names of the repository/repositories and accession number(s) can be found at: https://www.ncbi.nlm.nih.gov/bioproject/PRJNA849319).

## Abbreviations

TC: Total carbon
TN: Total nitrogen
TP: Total phosphorus
TK: Total potassium
AP: Available phosphorus
AK: Available potassium
OTUs: Operational taxonomic units
